# Effect of 1.5 wt% Copper Addition and Various Contents of Silicon on Mechanical Properties of 1.7102 Medium Carbon Steel

**DOI:** 10.3390/ma14185244

**Published:** 2021-09-12

**Authors:** Pavel Salvetr, Aleksandr Gokhman, Zbyšek Nový, Petr Motyčka, Jakub Kotous

**Affiliations:** COMTES FHT a.s., Prumyslova 995, 334 41 Dobrany, Czech Republic; aleksandr.gokhman@comtesfht.cz (A.G.); zbysek.novy@comtesfht.cz (Z.N.); petr.motycka@comtesfht.cz (P.M.); jakub.kotous@comtesfht.cz (J.K.)

**Keywords:** medium carbon steel, mechanical properties, microstructure, tempering, carbides, dilatometry

## Abstract

Requirements for mechanical properties of steels are constantly increasing, and the combination of quenching and tempering is the method generally chosen for achieving high strength in medium carbon steels. This study examines the influence of various silicon contents from 1.06 to 2.49 wt% and the addition of copper (1.47 wt%) on the behavior of 1.7102 steel starting with the as-quenched state and ending with the tempered condition at the temperature of 500 °C. The microstructure was characterized by SEM and TEM, the phase composition and dislocation density were studied by XRD analysis, and mechanical properties were assessed by tensile and hardness testing, whereas tempered martensite embrittlement was assessed using Charpy impact test and the activation energy of carbide precipitation was determined by dilatometry. The benefit of copper consists in the improvement of reduction of area by tempering between 150 and 300 °C. The increase in strength due to copper precipitation occurs upon tempering at 500 °C, where strength is generally low due to a drop in dislocation density and changes in microstructure. The increasing content of silicon raises strength and dislocation density in steels, but the plastic properties of steel are limited. It was found that the silicon content of 1.5 wt% is optimum for the materials under study.

## 1. Introduction

1.7102 steel is a medium carbon steel alloyed with some amount of chrome and silicon. This steel reaches both high strength and toughness and thus satisfies the requirements for spring materials or for other heavy-duty structural parts [1,2]. The heat treatment of medium carbon steel includes quenching followed by tempering or partitioning [3]. The typical as-quenched structure of medium carbon steels is lath martensite. The high strength of lath martensite is mainly associated with strengthening by carbon in interstitial solid solution, rearrangement of carbon atoms during and after quench, grain boundary strengthening, and dislocation strengthening [1,4,5]. On subsequent tempering, transition iron carbides (hexagonal ε-carbides and orthorhombic η-carbides) first form during low-temperature tempering (70–240 °C). The second transformation involves decomposition of retained austenite (200–300 °C), and the third tempering stage is characterized by the transformation of ε- or η-transition carbides to the more stable orthorhombic θ-cementite (200–450 °C) [6,7,8] when the silicon content is less than 2 wt%, while the monoclinic χ-carbides can also be formed before θ-cementite in steels alloyed with 2 wt% of silicon [9]. Mechanical properties, such as yield strength, tensile strength, and hardness, usually decrease during tempering. However, toughness and elongation increase with increasing tempering temperature [10,11,12,13]. In medium carbon steels alloyed with chrome and silicon, increasing strength was found for tempering at 350 °C [14], whereas alloying with manganese and silicon causes this effect in the tensile test temperature range from 200 to 250 °C [3]. In both studies [3,14], the increase in strength during tempering results from ε-carbides dispersed in the martensite matrix. The latter is provided by silicon alloying, which restricts cementite precipitation by forming a Si-rich layer around the growing cementite particles in the martensite matrix [15,16,17].

Enhancement of strength and hardness through copper precipitation strengthening was confirmed in low alloyed low carbon steels [18,19,20] as well as in medium carbon steels [7,12,21]. Tempered martensite embrittlement (TME) in martensitic steels was studied in [22,23,24]. It was found that TME relates to formation of platelet cementite, which replaced ε-carbide [22]; formation of thin carbide film along PAG boundaries [25]; decomposition of retained austenite (RA) between martensite laths into cementite and ferrite [23]; and segregation of impurities (such as phosphorus) along prior austenite grain (PAG) boundaries during austenitization [24].

This work investigates the evolution of mechanical properties of 1.7102 medium carbon steel and its variant with an addition of 1.47 wt% of copper and various contents of silicon from 1.06 to 2.49 wt%. All the steels were quenched and tempered between 100 and 500 °C. The effects of copper and silicon content on mechanical properties were determined by tensile testing and hardness and toughness measurement. The TME mechanism was discussed. Microstructure was observed using an electron microscope, and phase analysis and dislocation density measurement using X-ray diffraction were performed. The activation energies for the formation of transition iron carbides and cementite were measured by nonisothermal dilatometry. The results for 1.7102 steel modified by copper and silicon additions are compared to the properties of 1.7102 steel stated in the ISO 8458-1 standard [26].

## 2. Materials and Methods

### 2.1. Materials and Heat Treatment

The medium carbon steel BX with a similar chemical composition to steel 1.7102 according to ISO 8458-1 and four 1.7102 variants with different levels of copper and silicon were investigated. Their chemical compositions are given in Table 1. They were determined using Q4 TASMAN optical emission spectrometer (Bruker Elemental GmbH, Kalkar, Germany). The steels were melted in a vacuum induction furnace and cast into 45 kg ingots. The ingots were brought to 1050 °C and hot-rolled to a thickness of 14 mm and air-cooled. Normalization annealing was carried out at 850 °C for 40 min. Cylindrical samples 120 mm in length and 13 mm in diameter were manufactured. Thermal treatment of the samples consisted of quenching and tempering. The quenching temperature was 900 °C with a soak time of 20 min. The samples were quenched in oil at room temperature and then tempered between 100 and 500 °C for 120 min.

### 2.2. Mechanical Properties

Mechanical properties were determined by tensile, hardness, and Charpy impact testing on samples after quenching and tempering at 100, 150, 200, 250, 300, 350, 400, and 500 °C for steels B and BX and after tempering at 200, 300, 400, and 500 °C for A, C, and D steels at room temperature. Three tensile specimens were tested for each heat treatment condition. The tensile specimens were 8 mm in diameter and 50 mm in length. Quasistatic tensile tests were performed according to ČSN EN ISO 6892-1 at a rate of 0.75 mm/min using a Zwick Z250 testing machine with a 250 kN capacity (ZwickRoell GmbH & Co. KG, Ulm, Germany). Ultimate tensile strength (UTS), yield strength (YS), percentage elongation after fracture (elongation A_5_), and reduction of area (Z) were determined. Charpy V notch impact test was conducted at room temperature for three samples for each treatment route using Charpy pendulum WPM PSd 300 J (Kögel Werkstoff- und Materialprüfsysteme GmbH, Wachau, Germany) according to ČSN EN ISO 148-1. Charpy V notch specimens were prepared with 55 × 10 × 5 mm dimensions and 2 mm V notch depth. Hardness testing was carried out on a Vickers hardness tester (Struers Durascan 50, Copenhagen, Denmark) using a diamond indenter under the load of 10 kg for 10 s (ČSN EN ISO 6507-1) 10 times for each sample.

### 2.3. Microstructure Observation

Microstructural characterization was performed with scanning electron microscope (SEM) JEOL IT 500 HR (JEOL, Tokyo, Japan) in the longitudinal direction of specimens. Metallographic samples were prepared by grinding and polishing, and the microstructure was revealed by etching with 2% Nital reagent.

A transmission electron microscope (TEM) JEOL JEM 2200FS (JEOL, Tokyo, Japan) equipped with an energy dispersive X-ray spectrometer (EDS) was used for a more detailed analysis of microstructure and identification of transition carbides using selected area electron diffraction (SAED). Thin foils were prepared using the twin-jet electropolishing method (Fishionne Electropolisher M220) in the solution of perchloric acid (5 mL) and methanol (95 mL) at −50 °C.

### 2.4. X-ray Diffraction

X-ray diffraction (XRD) analysis was carried out using BRUKER D8 DISCOVER diffractometer with Cu K_α_ radiation (wavelength, λ = 0.15406 nm). Diffraction patterns were collected in the 2theta range from 30 to 120° with a step size of 0.02° and exposure time of 0.75 s/step. The retained austenite volume was characterized by Rietveld refinement method using Topas software. The dislocation density, *ρ*, was determined by modified Williamson–Hall (WH) method [27,28] from the full width at the half maximum (FWHM) and the diffraction angle of all peaks (101), (200), (211), (202), and (310), using Equation (1):

(1) $$∆K≅\frac{0.9}{D}+{\left(\frac{\pi M^{2}b^{2}}{2}\right)}^{\frac{1}{2}}\rho^{\frac{1}{2}}K{\bar{C}}^{1/2}$$

where $K=\frac{2sin\theta }{\lambda }$ and $∆K=\frac{2cos\theta \Delta \theta }{\lambda }$. Here, *Δθ* and *θ* represent the FWHM and diffraction angle, respectively; *b* = 0.248 nm is the magnitude of the Burgers vector; *M* is a constant depending on both the effective outer cut-off radius of dislocations and the dislocation density; ${\bar{C}}^{1/2}$ for the crystallographic plane (hkl) can be written as [27]:

(2) $${\bar{C}}^{1/2}={\bar{C}}_{h00}\left(1-qH^{2}\right)$$

where *q* is a parameter indicating the dislocation character in the sample and ${\bar{C}}_{h00}$ is a constant corresponding to the elastic constants of the material [27]. *H^2^* is represented as [27]:

(3) $$H^{2}=\frac{h^{2}k^{2}+h^{2}l^{2}+l^{2}k^{2}}{h^{2}+k^{2}+l^{2}}$$

The surface of samples for determining the FWHM was etched with hydrochloric acid for 120 s to remove the surface layer that had formed during polishing in order to minimize errors that might have originated from the polishing process.

### 2.5. Dilatometry

Two push-rod dilatometers, one with samples heated in a furnace for heating rates of less than 0.1 K/s and the other a quenching dilatometer with induction heating for other heating rates, were used to record the length reduction due to tempering. Nonisothermal tempering was performed using continuous heating with heating rates *β* = 0.005, 0.05, 0.5, 5, and 50 K/s. The activation energy *E* for the precipitation of carbides in the steels was calculated for the two stages of tempering using an approximate expression from [29]:

(4) $$Ln\frac{\beta }{T_{i}^{2}}=-\frac{E}{RT_{i}}+Ln\frac{RK_{0}}{E}$$

Here, *K_0_* is frequency factor, *R* is the gas constant, and *T_i_* indicates the inflection point in the dilatometer records obtained at various heating rates.

## 3. Results

### 3.1. XRD

**Figure 1:**
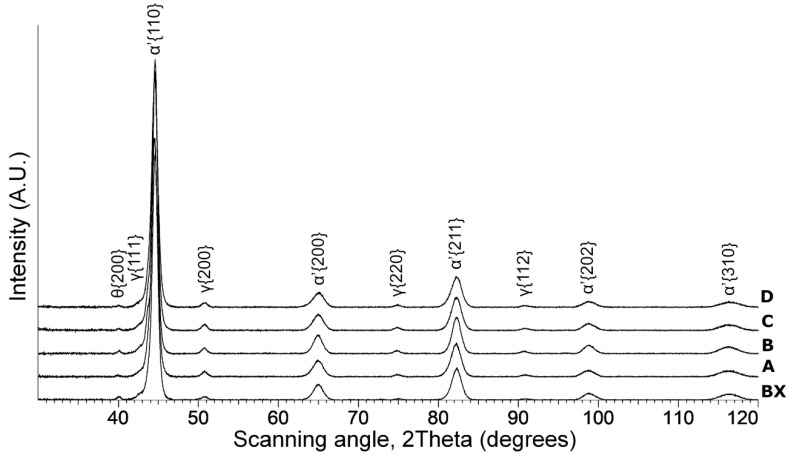
XRD patterns of the medium carbon steels with various contents of silicon and copper after quenching.

XRD patterns for the quenched steels are presented in Figure 1. Diffraction peaks of α’ martensite were detected. A weak peak of θ cementite was found as well. Retained austenite (RA) was detected (8.2%, 5.3%, 8.2%, 9.6%, and 8.1% for steels A, BX, B, C, and D, respectively). This is in line with the relatively low martensite start (*M*_s_) temperatures: 308.3, 316.9, 301.2, 296.0, and 292.2 °C for steels A, BX, B, C, and D, respectively. The *M_S_* temperatures were calculated using the following equation [30]:

(5) Ms (°C)=545−330C−23Mn−14Cr−13Ni−7Si+2Al+7Co−5Mo−13Cu (wt%)

The tempering at 500 °C for 120 min results in complete RA decomposition for all the steels under investigation.

**Figure 2:**
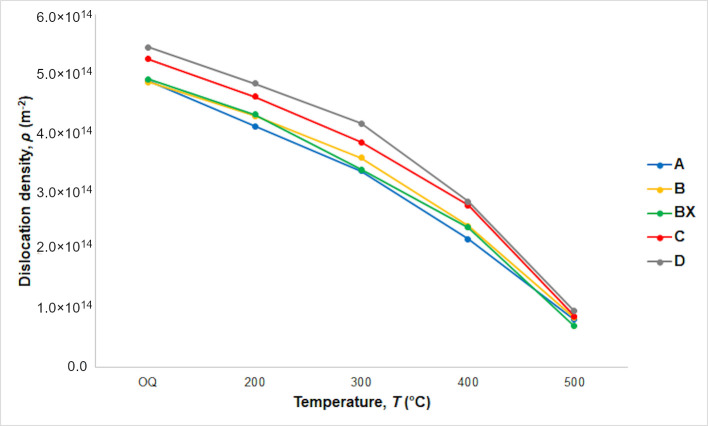
The dislocation density of quenched and tempered steels A, B, BX, C, and D.

The dislocation density, *ρ*, after quenching and subsequent tempering, as determined using XRD data in accordance with [27,28], is shown in Figure 2.

### 3.2. Dilatometry

**Figure 3:**
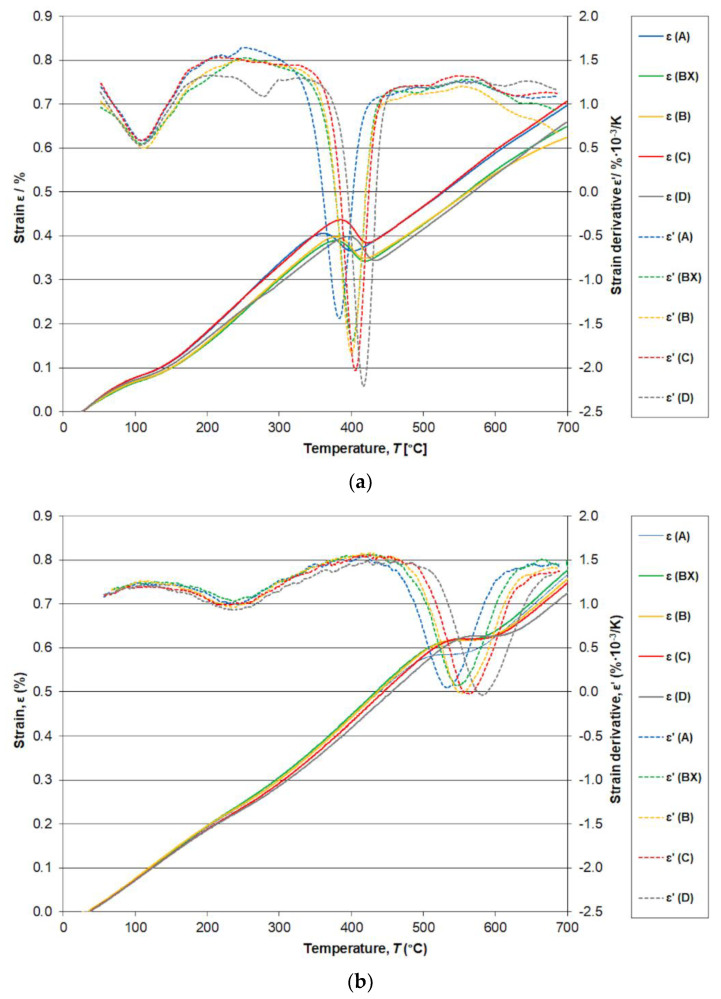
Thermal strain (*ε*) and its derivative (ε’) for steels A, BX, B, C, and D hardened from 900 °C and tempered using heating rate *β* = 0.005 K/s (**a**) and *β* = 50 K/s (**b**).

Dilatometry records of nonisothermal tempering in the form of thermal strain (*ε*) and strain derivative (*ε*’) dependences were determined for the steels at heating rates *β* of 0.005, 0.05, 0.5, 5, and 50 K/s. Figure 3 shows the dilatometry results for *β* = 0.005 K/s and *β* = 50 K/s. Two contractions are found in the dilatometry heating curves for the steels. The temperatures for inflection points, *T*_i_, for the first and second contraction are presented in Table 2 and Table 3. The inflection point for the first tempering stage indicates the precipitation of transition iron carbides, whereas the inflection point for the second tempering stage indicates the precipitation of cementite. A very significant effect of silicon content was found for the inflection points of cementite precipitation and less so for transition iron carbides. Higher Si content shifts the cementite formation to higher temperatures, and at the same time, the transformation gains in intensity. Higher heating rates shift the inflection point of strain for both stage I and stage II tempering to a higher temperature. At the same time, the reaction passes in a broader temperature range by higher heating rates. The activation energy *E* values for the steels calculated using Equation (4) are presented in Table 4.

### 3.3. Mechanical Tests

#### 3.3.1. Effect of Copper Addition

**Figure 4:**
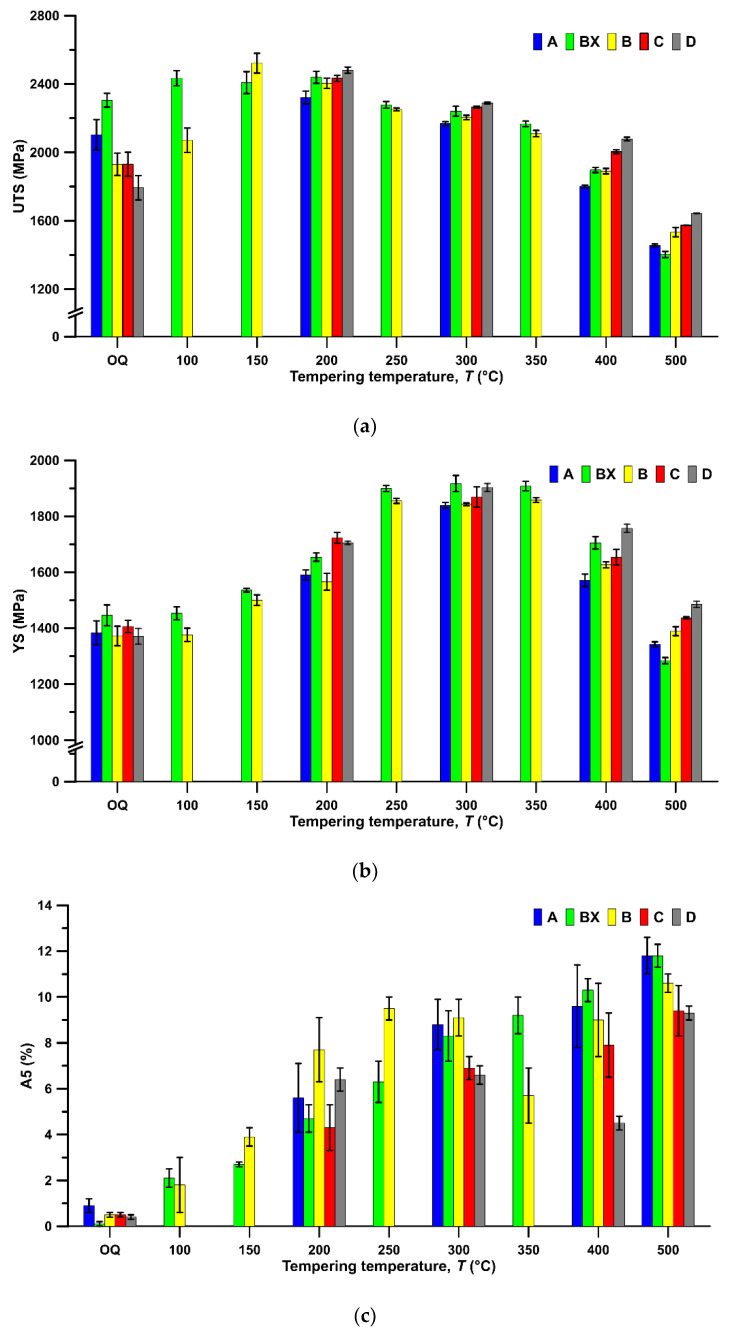

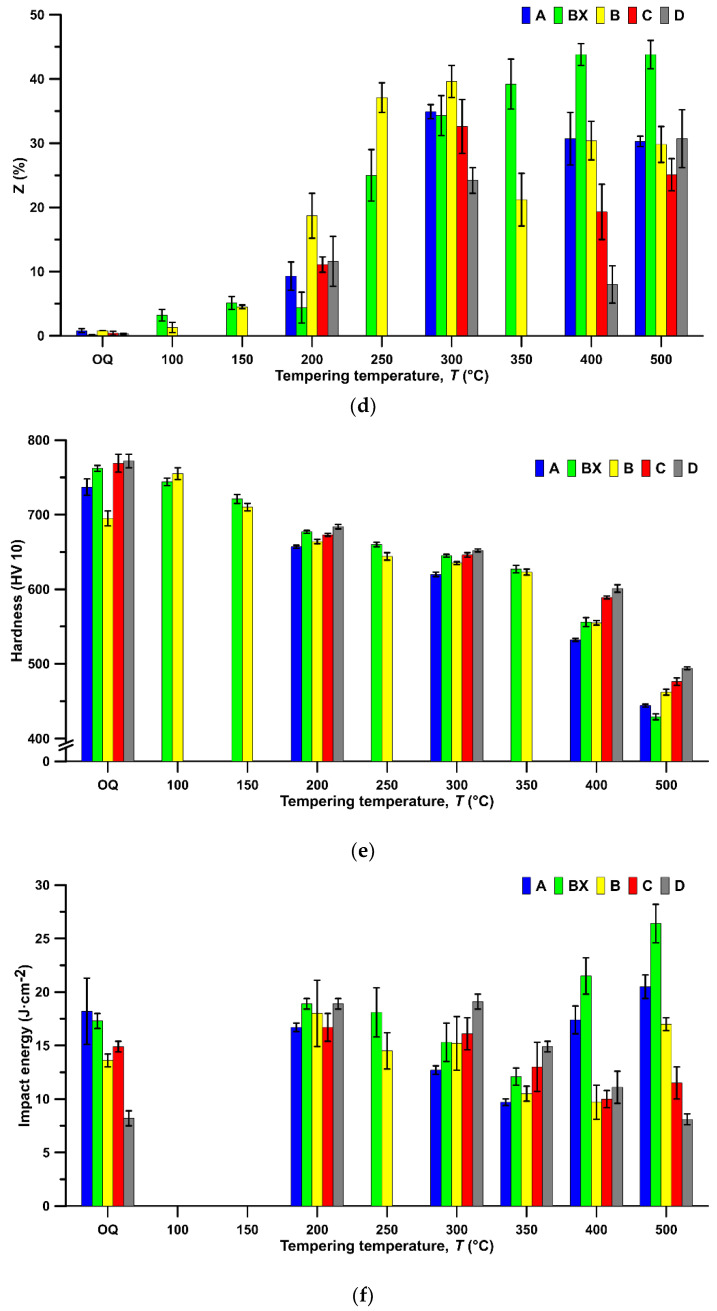
Mechanical properties of samples after quenching and tempering up to the temperature of 500 °C. (**a**) UTS—ultimate tensile strength; (**b**) YS—yield strength; (**c**) A5—elongation; (**d**) Z—reduction of area; (**e**) hardness; (**f**) Charpy impact energy of samples after quenching and tempering.

The results of the tensile test and Charpy impact test of steels B and BX, which illustrate the influence of copper alloying, are given in Figure 4. Yield strength and ultimate tensile strength of steel B are lower at lower tempering temperatures below 400 °C. There is one exception, at the tempering temperature of 150 °C, at which a slightly higher UTS of steel B was found. At the same time, copper has a beneficial effect on the elongation (A_5_) and reduction of area (Z) at tempering temperatures from 150 to 300 °C. However, the presence of copper in steel B impairs A_5_ and Z at higher tempering temperatures 350–500 °C. This is a very substantial difference that steel BX without copper alloying does not reveal an elongation decrease (A_5_) and reduction of area (Z). Both parameters increase continually with the increasing tempering temperature. Tempering at 500 °C brings additional strengthening by copper in steel B. Hardness is in good agreement with the results of the tensile test. Either the hardness values are similar or the hardness of steel B is slightly lower, up to the tempering temperature of 400 °C. At the tempering temperature of 500 °C, the hardness of steel B exceeds the hardness of steel BX. Charpy impact energy of steel BX after quenching and tempering, regardless of the tempering temperature, exceeds that for steel B, most notably at 500 °C.

#### 3.3.2. Effect of Silicon Content

A remarkable effect of silicon was found to occur just after quenching. As Figure 4 shows, YS and UTS increase continuously with increasing content of silicon in steels A, B, BX, C, and D. This occurs regardless of the tempering temperature, in the range of 200–500 °C. The difference in YS between steels A and D expands at the higher tempering temperatures, 400–500 °C. A similar trend is found with UTS. The impact of the silicon content on elongation and reduction is adverse, leading to their decrease, particularly up to the tempering temperature of 400 °C. The joint effect of copper and silicon in the steel promotes tempered martensite embrittlement (TME). The local maximum of elongation and reduction of area are found at the tempering temperature of 300 °C, beyond which a sharp decline occurs. The amount of this drop depends on the silicon content. In the low silicon steel A, the decrease of reduction of area is low and the elongation increases constantly during tempering. A completely different situation occurs in steel B alloyed with more silicon. The reduction of area and elongation decrease rapidly at the tempering temperature of 350 °C. Subsequent gradual improvement in these parameters occurs at higher tempering temperatures. The minima of reduction of area and elongation are found after tempering at 350 °C for steel B and at 400 °C for steels C and D. Tempering at 500 °C raises reduction of area and elongation in both high silicon steels C and D when compared to the tempering temperature of 400 °C. In steel D, the values exceed those in the steel C and reach similar levels to steels A and B. Hardness in A, B, C, and D steels increases with increasing silicon content, and the hardness difference between steels A and D ranges from 27 HV for the tempering temperature of 200 °C to 69 HV for the tempering temperature of 400 °C. The Charpy impact energy for steel A does not show a maximum at low tempering temperatures; instead, a minimum occurs at 350 °C. For steel D, there is a maximum at tempering temperatures of 200–300 °C, but no minimum at high tempering temperatures. Steels BX, B, and C have a maximum at 200 °C and a minimum at 350 °C.

### 3.4. Microstructure

**Figure 5:**
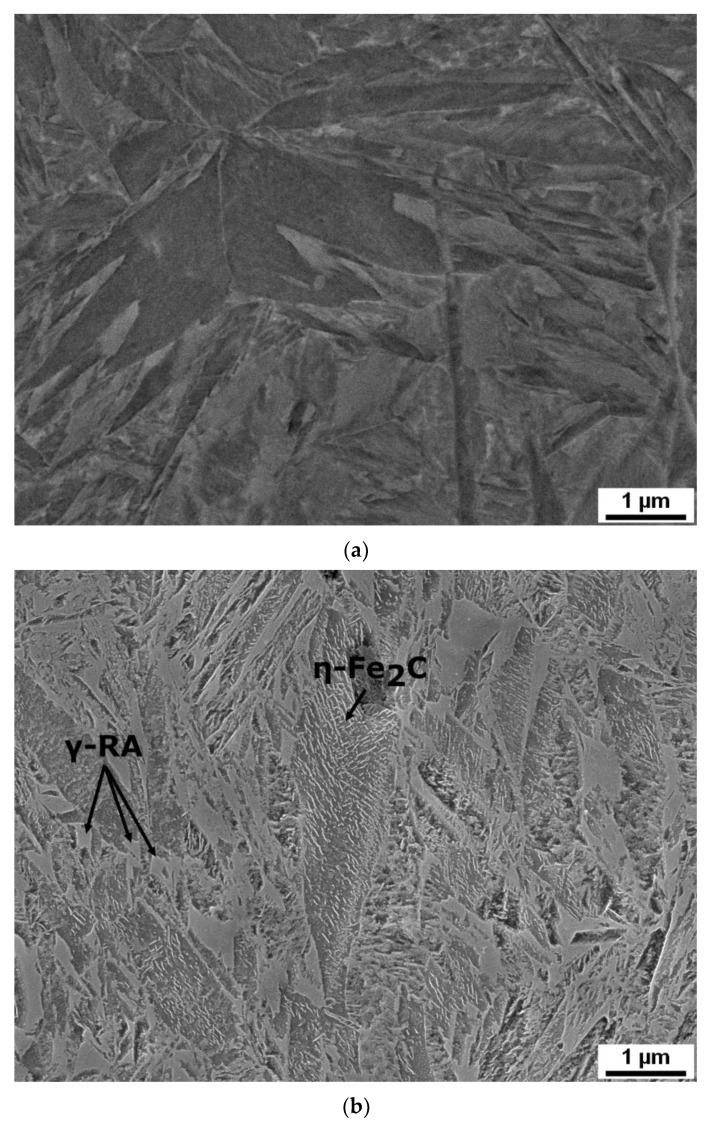

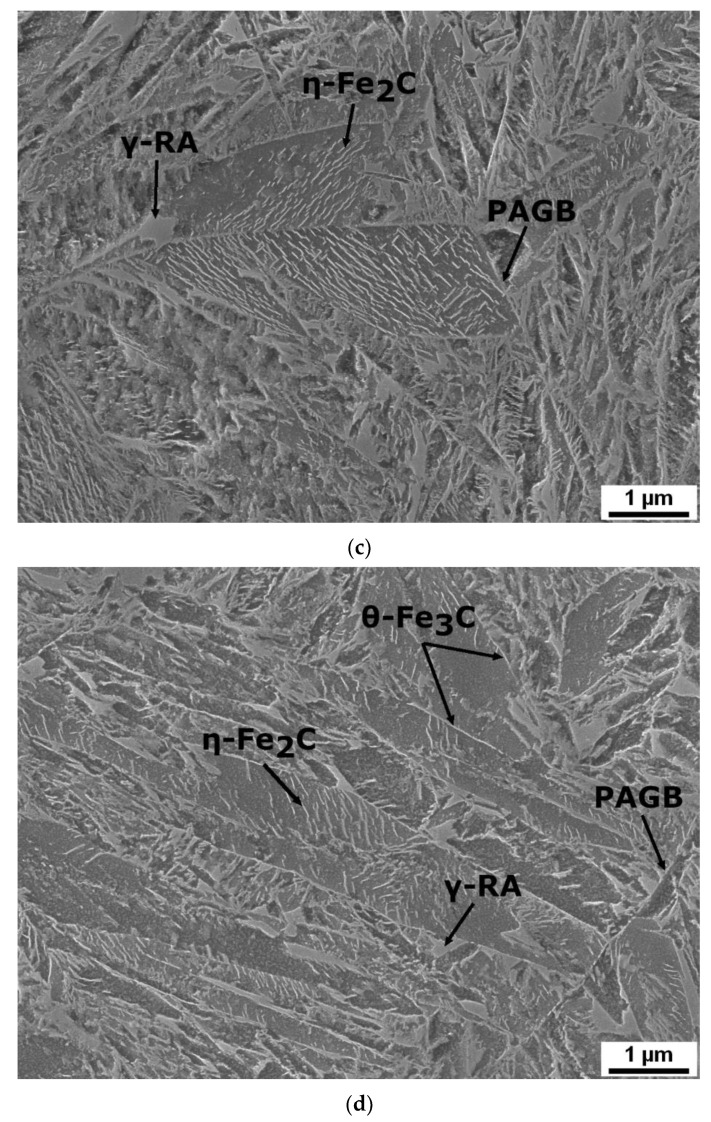

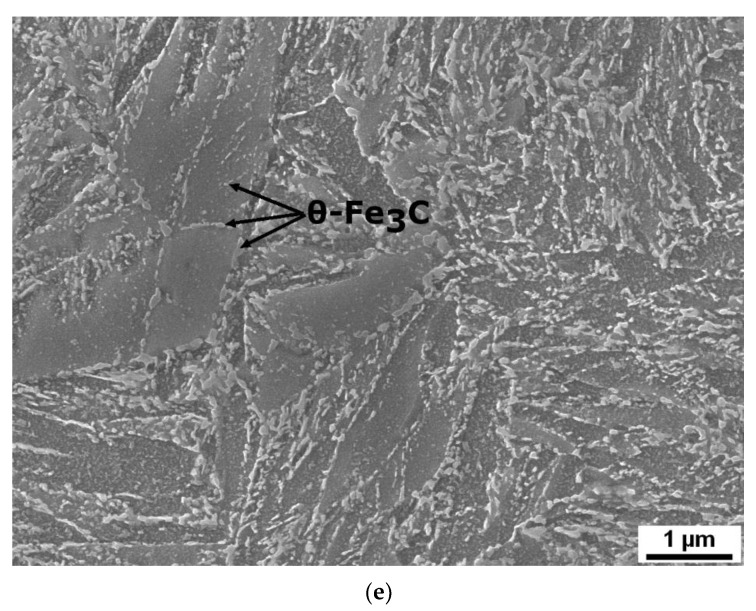
Development of microstructure of steel B during heat treatment (SEM images): after quenching (**a**) and tempering at 200 °C (**b**), 300 °C (**c**), 400 °C (**d**), and 500 °C (**e**). Martensitic matrix and RA are visible after quenching. The transition carbides and cementite form during tempering whereas the amount of RA decreases with the increasing tempering temperature.

**Figure 6:**
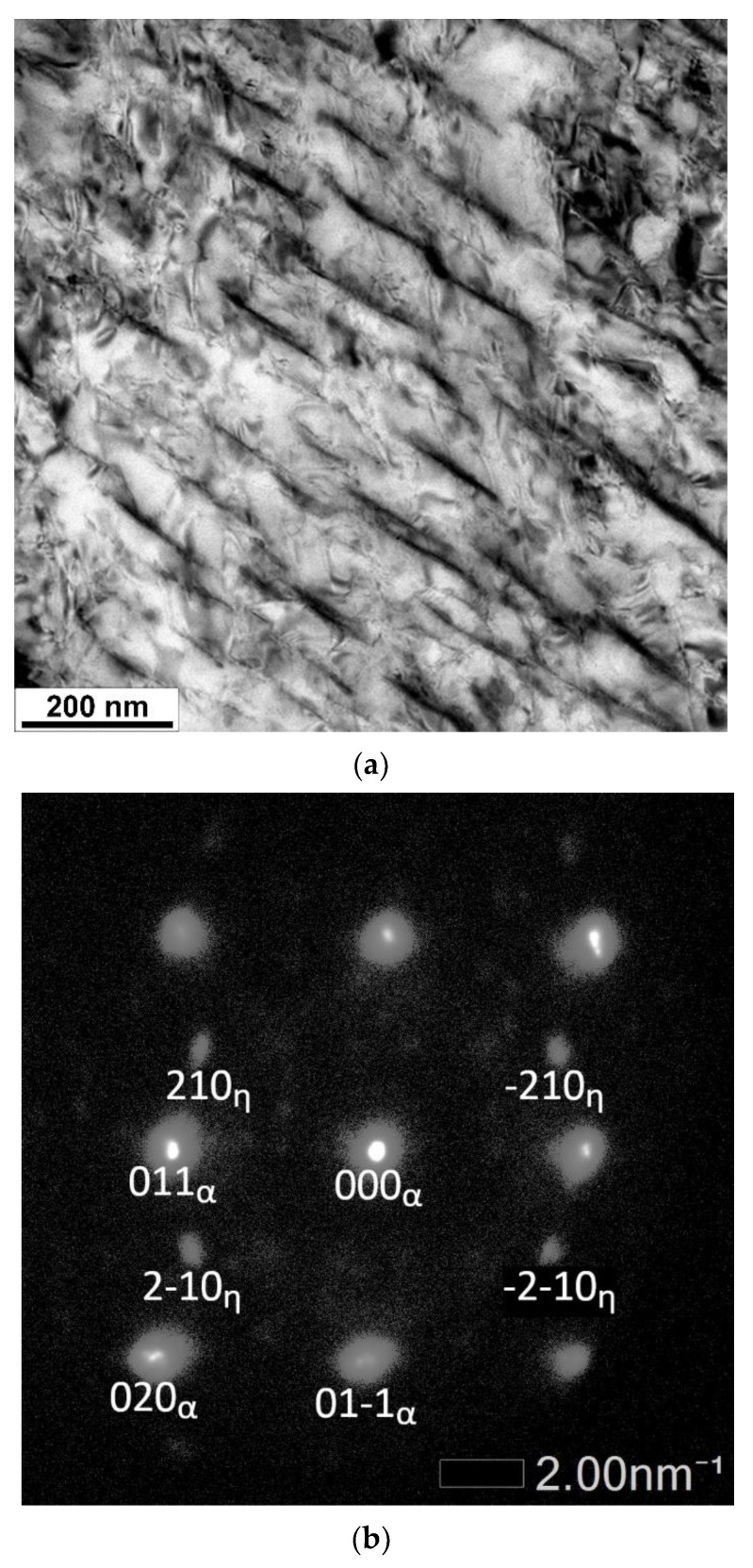
TEM micrograph of the η-Fe_2_C carbide (**a**) in steel B tempered at 200 °C and SAED pattern with zone axis [100] (**b**).

**Figure 7:**
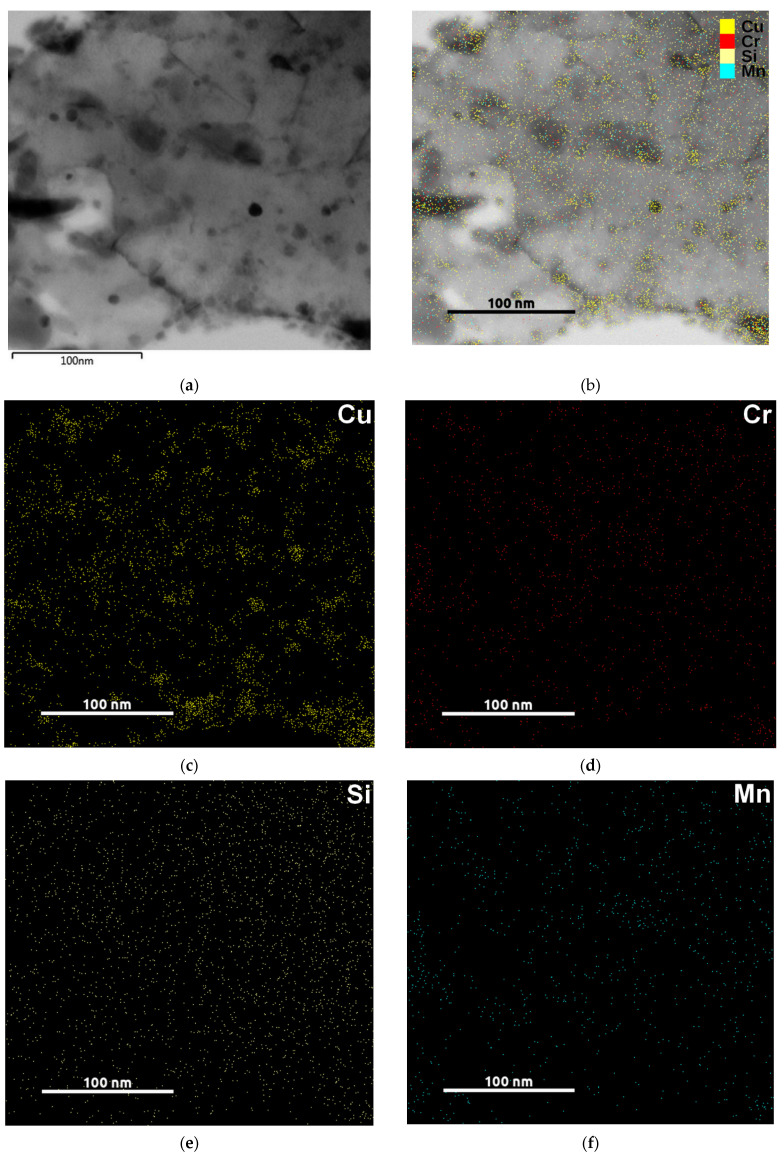
TEM-EDS map of chemical composition reveals the Cu-rich areas after tempering at 500 °C: TEM micrograph (**a**); TEM micrograph + EDS map of elements (**b**); Cu map (**c**); Cr map (**d**); Si map (**e**); Mn map (**f**).

SEM micrographs (Figure 5) document the evolution of microstructure during tempering: from the initial condition after quenching up to the tempering temperature of 500 °C. Similar changes occur in the microstructures of all the steels studied. Differences were only slight, such as the amount of retained austenite (RA). Hence, typical microstructural features occurring during heat treatment are only shown for steel B. After quenching, the microstructure consists of a martensitic matrix, prior austenite grains (PAG), and RA in various amounts, as determined using XRD measurement briefly described in Section 3.1. Rod-like transition iron carbides formed in the interior of martensite during tempering at 200 °C. These carbides are usually referred to as ε- or η-carbides with hexagonal or orthorhombic crystal structure, respectively, as described in [9,31,32]. In the material investigated here, only η-carbides were found (Figure 6). The amount of RA decreased gradually with increasing tempering temperature. The effects of different chemical compositions, mainly the various contents of silicon, were manifested at the tempering temperatures of 300 and 400 °C. Whereas the RA amounts significantly decreased in steels A, B, and BX, higher levels of RA (approximately 5%) remained stable up to the tempering temperature of 400 °C in high silicon steels C and D, and no RA was detected in any of the steels after tempering at 500 °C (see Table 5). Cementite films along martensite laths and apparent PAG boundaries were visible in the microstructure of steel B, as shown in Figure 5, after tempering at 400 °C. The presence of the cementite films along martensite laths and apparent PAG boundaries during tempering at 350–400 °C is in good agreement with previous studies [25,33] and with nonisothermal dilatometry data for cementite formation for the slowest heating rate β = 0.005 K/s (Table 3), as well as with the results of mechanical properties in Section 3.3 where TME was confirmed using Charpy impact test. The lowest toughness corresponds to the presence of the cementite film after tempering at 400 °C. Raising the tempering temperature to 500 °C led to spheroidization of cementite particles at lath and grain boundaries and precipitation of fine cementite particles in the interior of the martensitic matrix. At this tempering temperature, the Cu-rich clusters start to form in the microstructure of steel B as proven in the TEM-EDS map in Figure 7.

## 4. Discussion

### 4.1. Effects of Tempering Temperature

The experiment with varying tempering temperatures reveals the significant influence of this parameter on mechanical properties and microstructure just above 200 °C, even at short annealing tempering times (2 h). Key mechanical properties, such as ultimate tensile strength (UTS), yield strength (YS), elongation (A_5_), and reduction of area (Z) increase. This phenomenon is accompanied by microstructural changes, namely transition iron carbide precipitation and a decrease in dislocation density at the same time.

The YS shows a maximum between 250 and 350 °C whereas UTS has maximal values between 100 and 200 °C. It seems that the precipitation of transition carbides does not strongly affect UTS. Tensile strength responds more sensitively to the decrease in dislocation density. At the same time, YS is influenced by the precipitation more strongly.

The higher tempering temperatures cause a gradual decrease in both strength parameters. Accordingly, a strong increase in A_5_ and Z after quenching takes place during tempering up to 300 °C. Raising the tempering temperature above 300 °C delivers further improvement in plasticity only in the steel without Cu alloying. All the materials with Cu alloying show a drop in plasticity in the temperature range of 300–400 °C. One possible reason for this may be the segregation of Cu atoms at grain boundaries. During tempering above 400 °C, plasticity improved in all the materials.

The important challenge is to find the optimal tempering temperature. When maximum strength and satisfactory plasticity are sought, this temperature appears to be 300 °C for all the steels (Figure 4). This is consistent with the observed dependence of dislocation density on the tempering temperature (Figure 2) and the conclusions from dilatometry measurement that transition iron carbides, which improve mechanical properties, are formed already at 200 °C, whereas cementite, which could be harmful under certain circumstances, only replaces them on tempering at 400 °C (Figure 3). XRD measurement allows the conclusion that retained austenite (RA) does not completely decompose to cementite and ferrite at 300 °C (RA was found to be 5.6%, 5.6%, 7.4%, 9.6%, and 9.5% for steels A, BX, B, C, and D, respectively, when tempered at this temperature).

### 4.2. Effects of Si Content

Increasing silicon levels expand the temperature range for TME, followed by a loss of impact toughness. Therefore, silicon content is usually limited to no more than 2.2–2.6 wt% and, according to ISO 8458-1, it is allowed in the range from 1.2 to 1.6 wt%. In the present study, the effect of silicon content is investigated in the range from 1.06 to 2.49 wt% for 1.7102 steel.

It was found that dislocation density increases with increasing silicon content, but decreases almost 6 times with increasing tempering temperature.

A comparison of the data obtained on activation energies (Table 4) and data on copper-bearing steel with low silicon (0.21 wt%) [7], where E = 225 kJ/mol was found for the second stage, shows a significant effect of the addition of this element on the activation energy for cementite precipitation.

The activation energy was found to create a plateau for the second stage with an increase in the Si content above 1 wt% and a maximum at Si content of 1.5%.

This is consistent with [14,34], where the increase in hardness and tensile strength caused by increasing silicon content above 1 wt% was much larger than that from 1.8 to 2.44 wt%; i.e., the strengthening effect owing to the silicon addition saturates at about 1.8–2.5 wt%.

This is also confirmed by the nonmonotonic change in UTS depending on the Si content in steels A, B, C, and D tempered at 400 °C (see Figure 4).

Steel B (silicon content 1.54 wt%) has the best combination of strength and plastic properties among the steels under investigation.

Generally, the effect of silicon on carbon steels in quenched and tempered states can be characterized by several features, which were described in [6,14,34,35,36] and confirmed in this work. A higher silicon content in steels causes (a) higher amount of RA after quenching, (b) delayed decomposition of RA and formation of cementite during tempering, (c) shift of the TME region to higher tempering temperatures, and (d) achievement of higher values of UTS and hardness.

### 4.3. The Influence of Cu Content

Clearly, copper benefits the reduction of area and elongation between the tempering temperatures 150 and 300 °C, but tempering embrittlement sets in suddenly at tempering temperatures of 350–400 °C. Both elongation and reduction of area decrease apparently after tempering above 300 °C. Similar findings were reported in [14,34]. Energy-dispersive X-ray spectroscopy (EDS) data on higher content of copper in small precipitates when compared to the matrix in steel B tempered at 500 °C (Figure 7) are consistent with the results of mechanical testing of steels B and BX. Strength and hardness of B and BX are close, but the values of steel BX exceed those of steel B up to the tempering temperature of 400 °C. At this tempering temperature, the UTS and hardness values are comparable. At a tempering temperature of 500 °C, steel B has higher YS and UTS by approximately 120 MPa and hardness by about 30 HV than BX due to copper precipitation confirmed by EDS.

### 4.4. Effects of Quenching Temperature

Chemical composition of steel BX is similar to 1.7102 steel. However, the elongation, contraction, and Charpy impact energy for steel BX exceed those for 1.7102 steel, whereas the ultimate tensile strength and yield strength are approximately the same for these steels (Table 6). This illustrates the advantage of increasing the quenching temperature to 900 °C as used in the present study. This phenomenon could be explained by the elevated contribution of grain boundary strengthening. When increasing the quenching temperature, the prior austenitic grains become larger while the lath width decreases as shown in [37]. Proposing the lath width as an effective grain size, the Hall–Petch effect causes the grain boundary strengthening to increase [37].

### 4.5. Discussion of Different Strengthening Contributions and Their Influence on Final Properties

The yield strength (YS) of martensitic steels with less than 0.6 wt% carbon is mainly provided by martensite laths and retained austenite (RA). The YS of austenite is much lower than the YS of the matrix. Therefore, with the small fractions of RA found in the present study, the contribution of RA to YS can be neglected [38].

According to the model [39] for the strength of lath martensite, its YS can be found as a sum of five contributions: lattice friction stress (1), solid-solution strengthening (2), precipitation hardening (3), dislocation accumulation (4), and the Hall–Petch effect (5). It is obvious that the contribution (1) does not change with the increasing tempering temperature. While a decrease in dislocation density due to the increasing temperature (Figure 2) diminishes the contribution (4) and at the same time the decreasing carbon supersaturation diminishes solid solution strengthening (2), the contribution (3) concurrently increases due to emerging carbides (firstly, transition carbides; secondly, cementite) (Figure 5). The contribution (5) changes with a change in effective grain size. Thus, according to the model [40], one can conclude that, upon tempering at low temperatures, the contribution (3) exceeds contributions (2 and 4), but after tempering above 300 °C, the loss of contributions (2 and 4) exceeds contribution (3) for the steels studied.

At the tempering temperature of 350 °C, a minimum in Charpy impact energy was found (Figure 4). It seems that mechanisms (b) and (c) of TME, which refer to the formation of carbide film along PAG boundaries and the decomposition of retained austenite (RA) to cementite, are responsible for this phenomenon. Mechanism (a) of TME, which is the transformation of transition iron carbides to cementite, can be rejected because the temperature of the corresponding inflection point in the dilatometry records exceeds 350 °C (Table 3). Mechanism (d) of TME, which refers to the segregation of impurities (such as phosphorus) along prior austenite grain (PAG) boundaries, is also rejected due to the chemical composition of these steels. The maximum of Charpy impact energy at 200 °C on the curve of the tempering temperature dependence (Figure 4) can be explained by the formation of transition iron carbides and the effect of silicon on their stability [40]. Steel A is the only one where this maximum was not found, which is due to its relatively low silicon content (1.06 wt%). One remarkable fact is that TME in the steel with Cu alloying occurs across a considerably broader temperature range when compared to materials without Cu and the TME region shifts to higher tempering temperatures with increasing contents of Si.

## 5. Conclusions

The effects of tempering temperature, copper addition, and various contents of silicon on mechanical properties of medium carbon 1.7102 steel were studied.

The maximum strength and satisfactory plasticity are achieved for all investigated steels by a tempering temperature of 300 °C. This is consistent with the observed dependence of dislocation density on the tempering temperature and the data of dilatometry measurements about iron carbides: transition iron carbides, which improve mechanical properties, are formed already at 200 °C, whereas cementite, which could be harmful under certain circumstances, only replaces them on tempering at 400 °C.

Copper was found to have a beneficial impact on reduction of area and elongation between the tempering temperatures of 150 and 300 °C, but, at the same time, tempered martensite embrittlement (TME) occurs upon tempering at 350–400 °C.

A plateau in activation energy for precipitation of cementite and strength was found to occur with an increase in the Si content from 1 wt% with a maximum at Si content of 1.5%. One can thus recommend not to raise the Si content above 1.5 wt% in an effort to improve mechanical properties.

The competition between a decrease in the yield strength (YS) due to a decrease in the dislocation density and a decrease of carbon supersaturation of martensite on the one hand and an increase in YS contribution due to carbide precipitation on the other hand leads to a maximum of YS at tempering temperature at 300 °C for 120 min for each silicon content.

The TME at 350 °C is caused by the formation of carbide film along PAG boundaries and the decomposition of the retained austenite into cementite and ferrite. The TME mechanism, which refers to the transformation of transition iron carbides to cementite, is rejected because the temperature of the corresponding inflection point in the dilatometry records exceeds 350 °C. The TME mechanism, which refers to the segregation of impurities (such as phosphorus) along prior austenite grain boundaries, is also rejected due to the chemical composition of the studied steels.

The maximum strength and, at the time, satisfactory plasticity of medium carbon steel 1.7102 alloyed with 1.5 wt% Cu are provided by tempering at 300 °C for 120 min when silicon content is 1.54 wt%.

## Figures and Tables

**Table 1:** Chemical compositions of the steels studied and 1.7102 steel, wt%.

Steel (wt%)	C	Si	Cu	Mn	Cr	Ni	Mo	Fe
A	0.56	1.06	1.47	0.67	0.77	0.055	0.014	Bal.
B	0.56	1.54	1.48	0.70	0.77	0.056	0.016	Bal.
BX	0.57	1.51	0.12	0.68	0.75	0.13	0.032	Bal.
C	0.57	2.04	1.47	0.72	0.77	0.056	0.016	Bal.
D	0.57	2.49	1.47	0.75	0.77	0.058	0.016	Bal.
1.7102	0.51–0.59	1.2–1.6	-	0.5–0.8	0.5–0.8	-	-	96.2–97.3

**Table 2:** Temperatures corresponding to inflection points (*T*i) at different heating rates (*β*) for stage I of tempering in the dilatometry data.

*β* (K/s)	A	BX	B	C	D
0.005	110 °C	111 °C	112 °C	109 °C	108 °C
0.05	145 °C	144 °C	133 °C	144 °C	143 °C
0.5	162 °C	160 °C	162 °C	160 °C	160 °C
5	196 °C	190 °C	189 °C	190 °C	194 °C
50	237 °C	237 °C	239 °C	234 °C	238 °C

**Table 3:** Temperatures corresponding to inflection points (*T*_i_) at different heating rates (*β*) for stage II of tempering in the dilatometry data.

*β* (K/s)	A	BX	B	C	D
0.005	381 °C	399 °C	399 °C	404 °C	414 °C
0.05	418 °C	430 °C	433 °C	442 °C	452 °C
0.5	447 °C	462 °C	466 °C	473 °C	486 °C
5	485 °C	496 °C	494 °C	510 °C	524 °C
50	535 °C	551 °C	556 °C	565 °C	583 °C

**Table 4:** Activation energy *E* of formation transition carbides in Stage I and cementite in Stage II calculated for the steels A, BX, B, C, and D, using Equation (4).

Stage	A	BX	B	C	D
I	(114 ± 7) kJ/mol	(116 ± 7) kJ/mol	(112 ± 5) kJ/mol	(116 ± 8) kJ/mol	(112 ± 6) kJ/mol
II	(257 ± 7) kJ/mol	(272 ± 10) kJ/mol	(268 ± 15) kJ/mol	(265 ± 9) kJ/mol	(261 ± 8) kJ/mol

**Table 5:** Retained austenite content (RA) determined by XRD measurements.

RA (%)	Tempering Temperature (°C)			
	200	300	400	500
A	8.5	5.6	0.0	0.0
BX	8.3	5.6	0.4	0.0
B	9.8	7.4	1.1	0.0
C	10.8	9.5	5.2	0.0
D	11.8	9.7	5.6	0.0

**Table 6:** Properties of 1.7102 steel according to ISO 8458-1 compared with steel BX.

Steel	Quenching Temperature (°C)	Quenching Medium	Tempering Temperature (°C)	YS (MPa) min.	UTS (MPa)	A(%) min.	Z (%) min.	Impact Energy for 20 °C (J) min.
1.7102	840–870	oil	400–450	1300	1450–1750	6	25	8
BX	900	oil	400–500	1705–1300	1897–1402	10.3–11.8	43.9–43.9	9.9–14.1

## Data Availability

The data presented in this study are available on request from the corresponding author.
